# Understanding the relation between medical students’ collective and individual trajectories: an application of habitus

**DOI:** 10.1007/s40037-016-0321-1

**Published:** 2016-12-15

**Authors:** Dorene F. Balmer, Michael J. Devlin, Boyd F. Richards

**Affiliations:** 1 Perelman School of Medicine, University of Pennsylvania, Philadelphia, USA; 2 Columbia University Medical Center, New York, NY USA; 3 Center for Education Research and Evaluation, Columbia University Medical Center, New York, NY USA

**Keywords:** Bourdieu, Habitus, Medical students, Qualitative research, Transitions

## Abstract

**Introduction:**

While medical educators typically attend to group trends, groups are made up of unique individuals. An exploration of Bourdieu’s concept of habitus, defined as a system of dispositions, may help medical educators think relationally about the collective trajectory of the group and the individual trajectory of each student.

**Methods:**

We built on our 4‑year, longitudinal study which reported how field, capital, and habitus worked together to explain how medical students, as a group, navigated transitions in undergraduate medical education. In this secondary analysis, we reviewed serial collections of narratives about students’ peak learning experiences in medical school (19 students, 5 narratives per student), concentrating on first-person representations of self. We then explored the relation between collective and individual trajectories in three illustrative cases.

**Results:**

The social space of undergraduate medical education harmonized students’ experience and helped explain the collective trajectory, as evidenced by students’ consistent reports of taking initiative and staying open-minded. But individuals were not totally harmonized. They had unique dispositions that influenced their ability to access valued resources and shaped their behaviour. For example, Emily consistently spoke of being driven by her own goals; Zach focused on meeting expectations of authorities; Hilary routinely oriented toward abstract medical knowledge.

**Discussion:**

Habitus provides a useful conceptual lens for thinking relationally about collective and individual trajectories of medical students. Our work may inform faculty as they seek to situate individualized learning within standardized curricula, and is a step toward researching transitions in medical training from a holistic perspective that includes, but is not limited to, individual trajectories.

## What this paper adds

This study uses Pierre Bourdieu’s complex concept of habitus as a lens for understanding the relation between collective and individual trajectories of medical students as they navigate transitions in undergraduate medical education. Our work may inform faculty who seek to situate individualized learning within standardized curricula. It is also a step toward researching transitions from a holistic perspective that includes, but is not limited to, an individual trajectories.

## Introduction

Medical educators often attend to group effects and act in ways that yield the greatest possible good for the greatest number of medical students. However, attending to the group comes with the risk of not attending to individuals who comprise that group. For example, if team-based learning is embraced by medical educators because it addresses a core competency in the medical school’s formal curriculum, i. e., teamwork, then team-based learning is adopted as a teaching strategy for all students, regardless of individual learning preferences and efficiencies.

Pierre Bourdieu’s Theory of Practice in general, and his concept of habitus in particular, reconciles a tendency to think in either/or terms; as applied to the team-based learning example, attending to either the collective or the individual [1–4]. Briefly, habitus is a system of durable, transposable dispositions that is shaped by one’s past and present circumstances, and that shapes one’s present and future [1, 5, 6]. Habitus provides a means of thinking relationally about objective, social structures and subjective, practical activity [2]. It also provides a means of thinking relationally about collective and individual trajectories [3]. To use Bourdieu’s terminology, collective and individual trajectories are neither totally coordinated nor totally independent. He writes,

Since the history of the individual is never anything other than a certain specification of the collective history of his group or class, each individual system of dispositions may be seen as a structural variant of all the other group or class habitus, expressing the difference between trajectories and positions inside or outside the class [1].

Habitus is not a standalone concept, but rather to be considered in concert with two related concepts: field and capital. A field is a structured, social space with its own rules and traditions that shape how individuals and groups interact for the purpose of acquiring resources recognized as valuable within that social space. These resources constitute capital. Individuals and groups rely on habitus to secure capital which, in turn, enhances their influence within a field [6]. In recent years, others in medical education have used Bourdieu’s Theory of Practice as a conceptual lens, but not focused on habitus [7–10]. For example, Brosnan [10] reported that the field of medical education in the United Kingdom was shaped by schools’ competition for different forms of capital, such that some schools oriented towards biomedical sciences and others towards clinical practice.

Building on these studies, we used Bourdieu’s Theory of Practice to interpret interview data derived from a four-year, longitudinal study of medical students [11]. We observed how the field of undergraduate medical education shifted from an academic context in the preclinical phase to a clinical context in students’ major clinical year. The field shifted back to an academic context as students focused on securing a residency position; however, this time the academic context had a competitive tone. When academics predominated the field in the preclinical phase, students, as a group, sought capital in the form of applicable medical knowledge and relations with attending physicians who could facilitate connections within the medical profession. When the clinical context prevailed in students’ major clinical year, they sought capital in the form of having a reputation for providing excellent care. Finally, as students prepared for residency selection, they sought capital in the form of test scores and letters of recommendation.

We also observed that to access capital within the shifting field of undergraduate medical education, students, as a collective, consistently relied on habitus oriented toward taking initiative and staying open-minded. For example, students took initiative by participating in extracurricular activities in the preclinical phase, thus forming relations with physicians who could ‘open doors’. They took initiative in the clinical phase by ‘pushing themselves’ to excel in clinical care and to be noticed by attending physicians. And as students prepared for residency selection, they took initiative by ‘making the most’ of opportunities to connect with reputable physicians who could write letters of recommendation. In sum, we reported how field, capital, and habitus worked together to help explain students’ transitions in undergraduate medical education.

Even upon publishing our primary study described above, we were intrigued by the interplay between group habitus, and habitus that seemed unique to individual students. On closer review of our data, we noticed that the stories students’ told about their peak learning experiences varied considerably across students, but were relatively stable within an individual student over the course of four years. We believed a focused exploration of habitus might help medical educators think relationally about collective trajectories (group habitus) and individual trajectories (individual habitus), and ultimately, could inform medical educators as they seek to attend to individual learners within standardized curriculum. Thus, we posed another research question in this secondary analysis, ‘How might the concept of habitus speak to the relation between collective trajectories and individual trajectories of medical students?’

## Methods

Details of our primary study have been published elsewhere [11]. As part of a longitudinal case study, we interviewed 22 medical students at Columbia University College of Physicians and Surgeons (USA) over their four years of training. The Institutional Review Board at Columbia University Medical Center approved this qualitative study. We obtained written, informed consent from medical students before interviews began.

In the primary data analysis, we inductively created codes (i. e., words that act as labels for important concepts and ideas), iteratively revised codes based on incoming data, and then clustered coded data into three broad categories: field, capital, and habitus. Regarding the last of these three, we routinely applied two codes (‘putting yourself out there’ and ‘staying open-minded’) to segments of data in which students talked about how they gained, or planned to gain, different forms of capital within the field of undergraduate medical education. Thus, we identified taking initiative and staying open-minded as habitus shared by this group of medical students.

In the interviews, we collected narratives about medical students’ peak experiences, which we defined as salient events in medical school. Peak experiences did not have to be related to the formal curriculum or even a positive experience. For this secondary data analysis, we reviewed peak experience narratives from the 19 students whom we interviewed three times in the preclinical phase (at 4, 9 and 16 months into a total of 48 months, or 4 years, of medical school) and twice in the clinical phase (at 22 and 34 months into medical school), representing 86% of students who participated in the primary study. We borrowed a novel approach to analyzing students individual habitus from longitudinal psychological research called I‑poems [12, 13]. In contrast to thematic analysis, which tends to focus on the holistic meaning, I‑poems trace change and continuity in an individual’s sense of self [13]. By listening to how students talked about themselves via I‑poems, we hoped to describe and analyze their individual trajectories.

**Fig. 1a,b:**
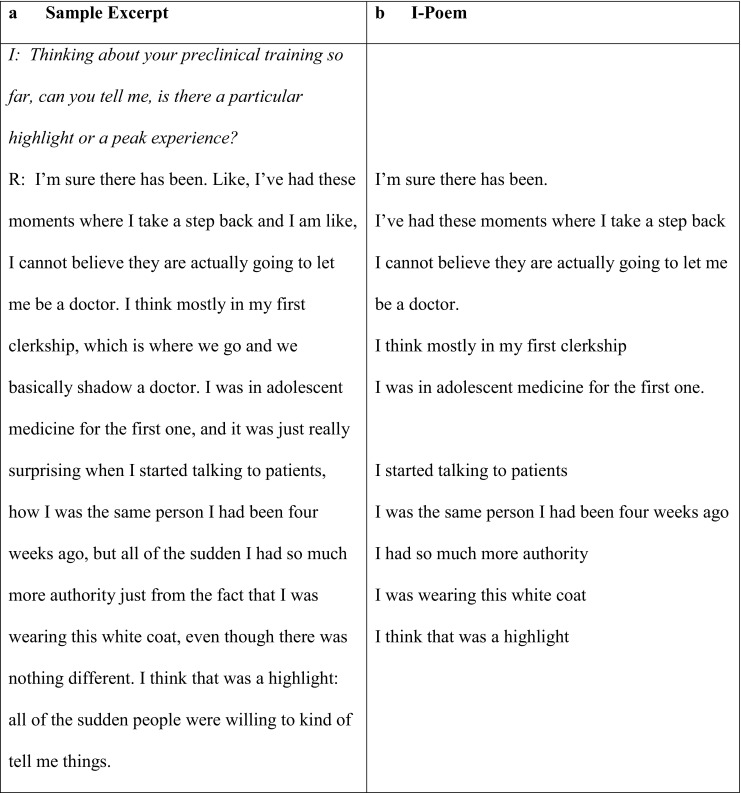
Sample excerpt from peak experience narrative and related I‑poem

Two of us (DB and MD) independently read and reread each of 95 peak experience narratives generated by 19 students in the secondary analysis (five narratives for each student). Concentrating on every first-person representation of self, (e. g., ‘I felt comfortable,’ ‘I was worried’), we transcribed first-person passages sequentially, and vertically, so that the selections read like a poem, (i. e., I‑poem). By way of illustration, Fig. 1a represents one peak experience narrative; Fig. 1b represents the I‑poem constructed from that narrative.

DB, MD, and BR met regularly as a team to review and refine their thinking about the individual trajectories reflected in each student’s collection of I‑poems. We invited a scholar well versed in social science theories, particularly Bourdieu’s Theory of Practice, to complement and sharpen our theory-informed analyses as we iteratively reflected on our understanding of habitus in general, and of the compilation of collective and individual trajectories in particular.

As a check on trustworthiness, we shared with eight medical students our description of the collective trajectory and their individual trajectory. We asked if, and how, our analysis resonated with their experiences. Their reactions and comments gave us confidence that our interpretations were aligned with their experiences. For this report, we chose three cases that we believe best enable us to think relationally about collective and individual trajectories. To maintain confidentially, we used pseudonyms and provide only general demographics.

## Results

Emily, Zach, and Hilary shared membership in the Class of 2014 at a prestigious medical school at a large research institution. This social space harmonized students’ experience of undergraduate medical education and helped explain the collective trajectory, as evidenced by their consistent reports of taking initiative and staying open-minded.

But Emily, Zach, and Hilary were not totally harmonized. They had unique dispositions that influenced their ability to access certain forms of capital and shaped their behaviour. Emily, for example, was older than most medical students, having worked for several years as a teacher and international volunteer. I‑poems provided a window into more nuanced understanding of individual trajectories, an understanding that could not be detected in differences in gender, education, or work-related experience (Table 1). In the following section, we present the stories of Emily, Zach, and Hilary in I‑poem format.

**Table 1:** Demographic information

	Emily	Zach	Hilary
Race	White-Caucasian	White-Caucasian	White-Caucasian
Citizenship	United States	United State	United States
Gender	Female	Male	Female
College education	Public school in the United States	Private school in the United States	Private school in the United States
Experience prior to medical school	Work experience	Research experience	No work or research experience

### Emily: achieving personal goals

Emily consistently talked about her personal goal of caring for the underserved, a goal crystallized through her years of international service before medical school. At the start of medical school, Emily referred to the intense preparatory work in the basic sciences as, ‘the crisis that happens in medical school’*. *Rather than succumbing to the crisis, Emily responded in line with her commitment to accomplish what she set out to do: get the training she needed to care for the underserved.

*I was unsure of my decision [to go to medical school].*

*I don’t live on campus.*

*I definitely did not feel like a part of the class.*

*I started to make some friends.*

*I said, ‘These are my people’.*

*I started to feel more comfortable.*

*I said, ‘I can do this.’*

*I took a few years to get to this point.*

*I think that [experience prior to medical school] changes how you view medical school.*

*I know exactly why I’m here*.

In her major clinical year, Emily stayed open to learning opportunities, reflecting group habitus. At the same time, she highlighted her unique history and drive to achieve her personal goal.

*I knew patients’ interactions would be a highlight.*

*I knew I was going to enjoy listening to them and learning them.*

*I didn’t expect all these people would be willing to take so much time to teach.*

*I’m coming into this a little bit older than other students.*

*I am less willing to waste time.*

*I’m interested in grabbing these learning opportunities.*

*I will regret it if I do not grab on [to them].*

In her last year of medical school, Emily spoke of her intended career in medicine and, like her classmates, recognized what it took to get into a competitive residency: academic rigour. Although she knew that it took initiative to obtain that capital, she reported that what drove her was her personal goal.

*I had a patient who was on the medicine service for two months.*

*I actually became her primary person.*

*I was rounding on her and everything.*

*I came out of my major clinical year really excited to start my career.*

*I had explored the things I was interested in.*

Emily’s unique dispositions were integral to her individual trajectory. And by virtue of class membership, the collective trajectory was also evident in her narratives.

### Zach: ‘Stepping up’ to expectations of authorities

Zach came to medical school singularly focused on a career in a highly competitive surgical subspecialty. Zach consistently spoke of ‘ambition’ and ‘stepping up’. And while all students spoke of taking initiative, Zach seemed particularly driven to meet expectations of external authorities. For example, Zach diligently positioned himself for success in his desired subspecialty, taking on roles that led him to work outside his comfort zone in order to gain necessary skills.

*I’ve always done things in my life that aren’t what I love the most.*

*I’ve just sort of done them.*

*I do them because I know they’re sort of good for me.*

Near the end of his preclinical training, Zach spoke of taking initiative to engage in extracurricular learning opportunities, even when those opportunities came at cost. For example, he travelled with the transplant team rather than study for the next day’s exam.

*I got to go on a transplant run.*

*I stepped up.*

*I’ll do anything to up my chances to do what I really want to do.*

*I got the call and I went.*

*I was pretty geared up for my first test*.

*I said to myself, ‘I would rather go on the transplant run.’*

*I’m always trying to pack it in*.

*I have always lived life to the fullest.*

Midway through his major clinical year, Zach spoke of his own progression relative to his peers. And meeting expectations for how quickly trainees should progress is what uniquely drove Zach.

*I could admit this guy to the hospital.*

*I can say I would not have done anything differently.*

*I saw this surgery intern who just came in fresh out of medical school.*

*I felt better about myself because, as a student, you are always on the lowest rung.*

*I see it as more attainable.*

*I know I can get there [internship].*

*I will someday arrive at this point.*

*I am becoming a doctor.*

After several arduous clinical rotations in his chosen subspecialty, Zach was frustrated by the failure of external authorities to validate his progress as an aspiring surgeon.

*I spend a lot of time standing around.*

*I think people appreciate my hard work, like I definitely help out.*

*I can help cannulate.*

*I know how to close all the way to the skin.*

*I kind of look over their shoulder most of the time.*

*I ask questions sometimes but a lot of it is me standing there.*

*I guess that is just the way it is.*

*I work very hard.*

*I do a lot but no one is teaching me.*

*I’m not going to cry about it*.

*I’m just trying to get into residency.*

*I’m smart enough.*

*I just stand around getting ignored a lot.*

*I just don’t understand sometimes.*

Despite his frustration, Zach continued to identify himself as a ‘competitive applicant’ in an equally competitive surgical subspecialty. His individual trajectory was constitutive of his habitus, but so too was the collective trajectory.

### Hilary: gaining abstract medical knowledge

Steeped in academics, Hilary initially found course work in medical school rather ‘cut and dry’. In contrast, what drove her was gaining abstract medical knowledge.

*I felt like everyone should know this stuff [pharmacology] but how great that I know it*!

*I understand this.*

*I’m going to be the one educating people about this.*

*I have always like chemistry.*

*I thought that pharmacology makes a lot of sense.*

In anticipation of her major clinical year, Hilary’s remarks reflected a relation between her individual trajectory, seeking abstract medical knowledge, and the collective trajectory, ‘doing it all’.

*I loved the renal lecture that integrated osmolality and volume.*

*I really liked that it was logical and made so much sense.*

*I tried to do all the shadowing opportunities that I could.*

*I really want to be a really good doctor.*

*I think one of the ways you do that is to shadow.*

*I think you do better in clerkships if you do that and if you understand the material.*

Consistent with her individual trajectory, Hilary believed that patients would change their behaviour if they simply had more knowledge about their illness, although this belief seemed to waver in her major clinical year.

*I wanted to try to believe the best in someone.*

*I would firmly believe that if I got someone to pledge they would exercise a couple of times a week, they would do it.*

*I probably didn’t, in retrospect, get them to change.*

*I really thought we were going somewhere.*

*I don’t know if it’s like that.*

Like her classmates, Hilary relied on habitus oriented toward taking initiative and staying open-minded. At the same, her drive for abstract medical knowledge was evident in her comments about preparing for residency selection.

*I have interviews with all five programmes in that city.*

*I have really high board scores.*

*I’ll get into the residency programme that I want.*

*I’ve had a lot of programmes tell me, ‘You’re going get into your number one choice.’*

Hilary’s dispositions, what set her apart from her classmates, contributed to her individual trajectory. But it did so in tandem with the collective trajectory of medical students.

## Discussion

The field of undergraduate medical education shaped the habitus of the students we studied, as evidenced by their tendency to take initiative and stay open-minded as they navigated transitions in medical school. And while the field of undergraduate medical education shaped habitus, it did not predict habitus. Each student had his or her own trajectory that was shaped by their individual past and would shape their individual future.

As Bourdieu reminds us, trajectories are never fully independent of others in the collective [1, 5]. Students in our study experienced the social space of undergraduate medical education in step with their classmates. But trajectories are not totally coordinated. In our study, students’ unique dispositions came into play as they transitioned from one phase of medical school to the next. In this way, individual trajectories were variants of the larger, collective trajectory.

### Implications for medical education research

Thematic analysis involves identifying patterns and regularities in qualitative data for the purpose of creating interpretive meaning [14]. In our primary study, thematic analysis helped us detect the collective trajectory. As a complement to thematic analysis, I‑poems helped us hone our ear to hear each student’s individual trajectory, a story we discerned by interviewing students throughout their four years of medical school. As others suggest, research which seeks to understand the interplay between the collective and the individual should capture and analyze data from different perspectives [15].

Our findings from this four-year longitudinal study add an important temporal dimension to cross-sectional studies about how students navigate transitions in medical school. Cross-sectional studies may capture the product of change, but not the process of change [16–19]. Our conceptually-driven, longitudinal study is a step toward researching transitions from a holistic perspective that includes, but is not limited to, individual trajectories [20].

### Implications for medical education practice

If habitus as a conceptual lens helps to ensure that the research focus is broader than the targeted focus of any given study, then our findings have important implications for the practice of medical education [3]. Students come to medical school with diverse histories and life experiences. However, they encounter curricula that valorize competencies – common and potentially reductive standards – for what every physician should be, should know, and should be able to do [21]. The concept of habitus may help medical educators think relationally as they grapple with questions such as, ‘How does one situate the unique learning preferences and efficiencies within a standardized, competency-based curriculum?’

When mentoring, faculty can support medical students in retaining their individual trajectories, thus honouring their experience of becoming a physician as a personal journey that is shaped, but not determined, by the social space of undergraduate medical education [22, 23]. At the same time, faculty can help students embrace shared experiences of the medical profession.

### Limitations

We recognize the potential limitations of our secondary analysis, and our limited scope when it comes to the complex concept of habitus. By focusing on habitus as a compilation of collective and individual trajectories, we did not focus on other aspects of habitus, such as its relation to agency or the interplay of past and present [3]. In our primary study, we did not specifically ask about habitus. However, by collecting peak experience narratives, we obtained students’ own identity claims, not prescriptive responses to direct questioning.

We interviewed a convenience sample of students from one institution, believing that those who volunteered for a longitudinal study would likely maintain their participation. None of the students we showcased were underrepresented minorities or were educated abroad; both could powerfully influence habitus. Our sampling strategy, however, does reflect the general makeup of Columbia University College of Physicians and Surgeons.

### Concluding remarks

In closing, we believe Bourdieu’s concept of habitus provides a useful conceptual lens for understanding the relation between collective trajectories (group habitus) and individual trajectories (individual habitus). In the end, we offer neither a prescription for balancing these trajectories, nor data that elevates either trajectory. Rather, we offer a reminder to hold collective trajectories and individual trajectories in our research and in our practice.
